# Relationship of Major Depression with Body Mass Index and Salivary Cortisol

**DOI:** 10.7759/cureus.6577

**Published:** 2020-01-06

**Authors:** Qudsia U Khan, Sehrish Zaffar, Abdul Mudabbir Rehan, Romana R Rashid, Huma Ashraf, Farida Hafeez

**Affiliations:** 1 Physiology, CMH Lahore Medical College and Institute of Dentistry, Lahore, PAK; 2 Pharmacology, CMH Lahore Medical College (NUMS), Lahore, PAK; 3 Pharmacology, D. G. Khan Medical College, Dera Ghazi Khan, PAK; 4 Physiology, Akhtar Saeed Medical and Dental College, Lahore, PAK; 5 Biochemistry, CMH Lahore Medical College (NUMS), Lahore, PAK; 6 Physiology, CMH Lahore Medical College (NUMS), Lahore, PAK

**Keywords:** cortisol, depression, body mass index

## Abstract

Introduction

Depression is one of the most incapacitating psychiatric diseases that disturb life of millions of people round the globe. Its major causes include stressful life events, bereavement, social abuses or certain biological and genetic factors with complex causal mechanisms. Higher salivary cortisol levels for a long period lead to dyslipidemias which increase body mass index (BMI), elevate adiposity and waist-to-hip ratio (WHR). Such individuals with high quartiles of BMI have considerably higher risk of major depressive disorder. The aim of this study was to establish a correlation between major depression, BMI and salivary cortisol.

Methods

This cross-sectional analysis was accomplished in the Physiology Department, Sheikh Zayed Federal Postgraduate Medical Institute, Lahore as well as in Punjab Institute of Mental Health, Lahore, Pakistan, over a period of six months. A total of 60 participants aged between 18 and 60 years were included in this study; they were divided equally into two groups as normal healthy individuals with no physical or mental illness and severely depressed groups. The patients were categorized as cases of severe depression on outdoor clinical assessment and further confirmed by ICD-10. Patient’s BMI was estimated by measuring height in meters (m) and weight in kilograms (kg), and then dividing weight with square height. Early morning saliva samples were collected. Estimation of cortisol levels in saliva was done through ELISA. SPSS version 20.0 (IBM Corp., Armonk, NY) was used to analyze the data and p ≤ 0.05 was considered statistically significant.

Results

The mean BMI in normal healthy group was 22.02 ± 4.21, while the mean BMI in severely depressive group was 24.64 ± 3.58. The difference was statistically significant (p = 0.012). The mean salivary cortisol level was significantly raised in patients with major depression (2.23 ± 1.69 nmol/L) in contrast to healthy normal individuals (1.46 ± 0.91 nmol/L), with p-value = 0.031.

Conclusion

BMI and depression has a very noteworthy correlation and there is a remarkable link between raised salivary cortisol, greater BMI and development of major depression.

## Introduction

Depression is one of the most incapacitating psychiatric diseases that disturb the life of millions of people round the globe [1]. Its major causes, which are often implicated, include stressful life events, bereavement, social abuses or certain biological and genetic factors with complex causal mechanisms [2]. Life complexities and tough schedules make depression 2.5 times more likely in stressed individuals. Epidemiological studies show that 80% of cases of depression are related to some emotional life event [3]. It manifests as painful symptoms of restlessness, worthlessness, hopelessness, pessimism and lack of interest in pleasure, culminating as suicidal thoughts and tendencies [4]. There is approximately 35% chance of its transferring towards the next generation [5].

The dysregulation of neurotrophic hormones and growth factors plays a crucial part in the pathophysiology of depression [6]. Various neural hormones including brain derived neurotropic factor (BDNF), angiogenic cytokines, vascular endothelial growth factor (VEGF) as well as salivary cortisol are perceived as important influencing elements in depression [7]. Salivary cortisol is a hormone secreted during stress. It affects blood glucose, blood pressure, immunity and lipoprotein metabolism [8]. The enzymatic functioning of lipoprotein lipase and insulin is reduced after the stimulation of salivary cortisol [9]. Consequently, it causes “Cushing's syndrome” also known as hyper salivary cortisolism, resulting in decrease in serum high density lipoprotein (HDL) and an increase in serum low density lipoprotein (LDL) [10].

In accordance with the National Institute of Health (NIH), the body mass index (BMI) is a key index for relating weight to the height. Overweight values of BMI are 27.8 and 27.3 for males and females, respectively. BMI of 30 or more is considered as obesity for either sex [11]. Higher salivary cortisol levels for a long span lead to dyslipidemia, which increases BMI, adiposity and waist-to-hip ratio (WHR) [12]. Meanwhile, the alterations in emotional and cognitive behaviors among kids and adolescents are also associated with raised level of adrenal steroids including dehydroepiandrosterone (DHEA) and salivary cortisol [13].

Several diseases like diabetes mellitus, hypertension, depression and heart diseases are found to be related to obesity and higher BMI. It is a common conception that people with obesity suffer with more psychological agony, which leads to development of anxiety [14]. According to a meta-analysis report, there is higher co-morbidity with anxiety, depression and obesity in adults and youth. Obesity raises the risk of depression and a reciprocal association exists between both of them. Hence, obesity and depression have overlapping pathophysiology [15,16].

Depression is a disabling problem and lot of antidepressant medications are available but the variability of their response on lots of individuals makes them uncertain for curing measures [17,18]. The response of these drugs is also influenced by the body weight which alters the pharmacodynamics and pharmacokinetic mechanisms [19]. Moreover, individuals of all ages with high quartiles of BMI have higher risk of depression [20]. Obesity is not only associated with anxiety, but also increases the incidence of somatic complications such as dyslipidemia, insulin resistance, arterial hypertension and diabetes mellitus [21,22]. Studies relating depression with salivary cortisol have not been done in Pakistan so far. Therefore, such correlation, if established, can serve to stratify high risk individuals and help in early diagnosis and management of such patients.

## Materials and methods

This cross-sectional analysis was accomplished in the Physiology Department of Sheikh Zayed Federal Postgraduate Medical Institute, Lahore as well as in Punjab Institute of Mental Health Lahore, Pakistan, over a period of six months. Institutional review board approval was obtained before commencement of the study. The participants were assured of anonymity and confidentiality, and verbal informed consent was taken from each participant. A total of 60 participants aged between 18 and 60 years were included in this study. They were selected from the outdoor department of Punjab Institute of Mental Health, Lahore.

On outdoor clinical assessment, the patients were categorized as cases of severe depression according to ICD-10 and DSM-IV criteria. Individuals’ data comprising name, gender, age, weight, height, BMI, educational status along with the overall physical wellbeing and history of depression in family was documented in a predesigned proforma. Detailed medical history was taken and physical examination of each subject was performed. The level of depression was assessed by the Beck’s Depression Inventory Questionnaire and was used as basis of inclusion into the study. According to Beck's scoring, depression is classified as mild, moderate and severe. Proformas were completed by inquiring the questions from the participants [23]. The subjects suffering from Cushing’s syndrome/disease, hyperaldosteronism and pregnancy were excluded. Patients taking antidepressants or exogenous steroid drugs were also excluded.

The participants fulfilling the inclusion criteria were added to the healthy group or the severely depressed group, on basis of Beck's scoring. Thirty participants were added in each group.

Patients’ height and weight were measured in meters (m) and kilograms (kg), respectively. BMI was estimated using the formula;

BMI = weight (kg) / (height in m)^2 ^ [11].

Early morning saliva samples were collected. Subjects were asked not to eat, drink or brush their teeth prior to sample collection. Before collecting samples, all the subjects were asked to rinse the mouth with normal saline. Saliva samples (4-5 ml) were collected in clean glass tubes and stored at -20°C for 24 hours. The samples were centrifuged at 20,000 rpm for 10 minutes, then transferred to Eppendorf tubes, properly marked and stored at -20°C for 30 days, till further analysis. Estimation of cortisol levels in saliva was done through ELISA [24]. ELISA Kit Accu Diag TM (Ref 7101-38) was used. The collected data was analyzed with SPSS version 20.0 (IBM Corp., Armonk, NY). Mean variance of cortisol level was established in accordance with few substantially fluctuant parameters such as age, weight, height, BMI as well as depression. Kolmogorov-Smirnov test was applied to check the normality of the variables. As the data was normally distributed, independent t-test was applied and p ≤ 0.05 was considered statistically significant.

## Results

The mean weight was 61.55 ± 11.61 kg in healthy normal group and 65.79 ± 10.92 kg in major depression group. The mean height was 1.67 ± 0.08 meters in healthy normal group and 1.63 ± 0.08 meters in major depression group. The mean BMI was 22.02 ± 4.21 in healthy normal group and 24.64 ± 3.58 major depression group. Mean difference between the height and weight of the healthy normal group and major depression group was insignificant, with p-value 0.056 and 0.151, respectively. However, mean difference between the BMI of the two groups was statistically significant, with p-value 0.012 (Table 1).

**Table 1:** Correlation of height, weight and BMI between the two observed groups * = p < 0.05

Variables		Minimum value	Maximum value	Standard deviation	Mean value	p-value
Height (m)	Healthy normal	1.52	1.85	0.08	1.67	0.056
	Major depression	1.55	1.83	0.08	1.63	
Weight (kg)	Healthy normal	42.90	99.80	11.61	61.55	0.151
	Major depression	40.80	86.70	10.92	65.79	
BMI	Healthy normal	16.20	35.20	4.21	22.02	0.012*
	Major depression	16.80	31.90	3.58	24.64	

In healthy normal group, the mean cortisol level was 1.46 ± 0.91 nmol/L, whereas in major depression group, the mean cortisol level was 2.23 ± 1.69 nmol/L. The mean level of salivary cortisol was significantly raised in patients with major depression in contrast with healthy normal individuals, with p-value 0.031 (Table 2).

**Table 2:** Assessment of salivary cortisol level in two observed groups * = p < 0.05

Salivary cortisol level (nmol/L)					
Study Groups	Minimum value	Maximum value	Mean value	Standard Deviation	p-value
Healthy normal	0.36	4.33	1.46	0.91	0.031*
Major depression	0.35	6.34	2.23	1.69	

## Discussion

Major depressive disorder is a devastating public health illness with acute social and individual costs, emotionally involved. There is substantial etiological and irrefutable heterogeneity, as one in every six persons suffers with this complex disorder in his/her life. Presence of cortisol levels in saliva is measurable in such patients and, perhaps, the disease progression is indicated by these markers [25]. A strong association between high BMI and major depression has been found in recent research. The results of one study clearly showed that depression and high BMI are interlinked [13]. Another study observed similar outcomes that BMI and depression are closely related; higher the BMI, greater the probability of depression [26].

Depression has been labelled as a prominent basis of disability in the entire world by the year 2020, according to World Health Organization [27]. A study conducted by Tashakori et al. has shown that there is a progressive and significant relationship between depression and BMI. Even after excluding familial depression, demographic risk factors and chronic physical diseases, the risk of development of depression keeps lingering, owing to extreme obesity. Body image dissatisfaction, social stigma and failed attempts at weight loss make obese women more susceptible to depression [28].

Another study highlights the predisposition to suicide in people with high BMI and depression. Obesity can induce depression by biological mechanisms like dysregulation of hormonal system. Depression makes an individual prone to unhealthy lifestyle and physical inactivity. Such factors further increase the risk of obesity and the viscious cycle goes on until a catastrophe like suicide takes place [29].

The potential association among depression and obesity has been recognized and frequently investigated due to great prevalence and the facts that they both are responsible of an increased risk for cardiovascular diseases. Such an association has been confirmed at the cross-sectional level. The present study emphasizes on the fact that presence of heavy amount of cortisol secretions in individuals with depression may indicate the role of hypothalamo-pituitary-adrenal (HPA) axis in predicting the development of disease and its prognosis. It is of foremost importance to take measures against the risk factors involved in the development of depression. The BMI can be improved by counselling and promoting lifestyle changes in mass population. Dietary intake should be monitored and healthy nutrition should be reinvigorated [30]. Persistent and high cortisol secretions lead to metabolic derangement that predisposes to obesity and worsen the depression in patients. Limitations of this study included small sample size due to scarcity of resources and personnel.

Salivary cortisol is emerging as a useful tool for early diagnosis of the disease. Currently, Pakistan lacks research on major depression and its relation with salivary cortisol secretion. The present study has highlighted a significant correlation of depression with BMI and salivary cortisol.Therefore, this study can help in identifying high risk individuals and their prompt therapeutic management can lead to improved clinical outcomes.

## Conclusions

Body mass index, salivary cortisol and depression has a definite correlation and salivary cortisol can be potentially used as a biomarker for early detection and management on major depression, in future.
